# The Influence of Temperature Disturbance on Space Inertial Sensors

**DOI:** 10.3390/s24216934

**Published:** 2024-10-29

**Authors:** Jia Hao, Fulong Wei, Xingyu Yan, Jinlong Ma, Zebing Zhou, Xiaobing Luo

**Affiliations:** 1 School of Energy and Power Engineering, Huazhong University of Science and Technology, Wuhan 430074, China; 2 MOE Key Laboratory of Fundamental Physical Quantities Measurement, Hubei Key Laboratory of Gravitation and Quantum Physics, School of Physics, Huazhong University of Science and Technology, Wuhan 430074, China

**Keywords:** TianQin program, inertial sensor, temperature perturbation, acceleration noise

## Abstract

The TianQin program is an independently proposed space-borne detection initiative from China. The inertial sensor, as a crucial component, is susceptible to disturbances from temperature fluctuations, the impact of which on acceleration measurements remains elusive. Therefore, a comprehensive analysis on the influence of temperature disturbance is necessary to avoid errors in acceleration measurement. In this study, we proposed a complete scheme for calculating the level of acceleration noise. Based on the traditional temperature control scheme of using a heat pipe, we developed a systematic heat transfer simulation model to obtain the temperature signals. These signals can then be converted into the frequency domain and used to calculate the acceleration noise level within the target bandwidth. Our results indicate that, with a temperature control of 0.13 K/Hz^1/2^@1 mHz, the largest contributions to acceleration noise come from the outgassing effect, radiometer effect, and radiative pressure effect, in descending order. The total noise peak is $3.6\times 10^{-12}\;m/(s^{2}\cdot {\mathrm{Hz}}^{1/2})$ at 1 mHz, which is more than three orders of magnitude higher than the TianQin target of $10^{-15}\;m/(s^{2}\cdot {\mathrm{Hz}}^{1/2})$. This study provides new avenues for evaluating measurement errors and solutions for the TianQin program.

## 1. Preface

The highly precise gravitational wave detection program is and will continue to be one of the most cutting-edge research topics in basic science for the foreseeable future. Einstein’s theory of general relativity once predicted the existence of gravitational waves [1]. And in 2016, the ground-based Laser Interferometric Gravitational Wave Observatory (LIGO) in the United States announced the first direct detection of gravitational waves generated by the collision of two black holes 1.3 billion light years away [2], confirming the existence of gravitational waves. Compared with ground-based detectors, space-borne gravitational wave detection can exclude an additional variety of ground noise interference, such as seismic noise and so on, resulting in higher sensitivity and the ability to capture signals at lower frequencies.

**Figure 1:**
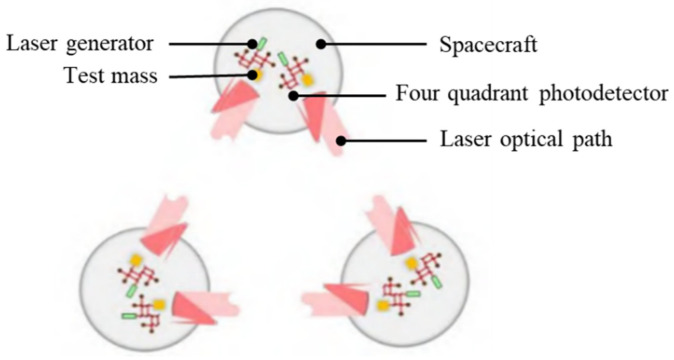
Schematic diagram of gravitational wave measurement in the low frequency band [7]. Relationship between laser interferometric measurement link and inertial sensor in TianQin Project, in which the gray part is spacecraft, the yellow part is the test mass, the green part is the laser, the black spot is the four-quadrant photodetector, and the red line represents the laser optical path.

The TianQin project, proposed independently by China, is a space-borne gravitational wave detection mission aimed at detecting gravitational waves in the middle and low frequency band (1 mHz~0.1 Hz) [3]. The measurement principle is shown in Figure 1, and the gravitational wave detector is a triangular laser interferometer system consisting of three spacecraft orbiting the Earth. When gravitational wave signals pass through, the laser interferometry system senses the tiny acceleration changes between the test masses suspended inside the inertial sensors and uses these changes to obtain information about gravitational waves [4], allowing for a deeper study of the cosmic evolutionary process. Gravitational wave signals are extremely weak at low frequencies. In order to achieve high-precision detection, the TianQin mission requires that the residual acceleration noise not exceed $10^{-15}\;m/(s^{2}\cdot K^{1/2})$ [5]. Therefore, the inertial sensors need to eliminate the interference of various physical noises, such as magnetic field perturbations, electrostatic perturbations, temperature perturbations and so on. Among them, temperature perturbations mainly comes from Earth flux, solar radiation changes, and power consumption changes of onboard components, and play an important role in the error evaluation of the TianQin program [3,6].

Noise caused by temperature perturbation in inertial sensors mainly includes the radiometer effect, radiation pressure effect, outgassing effect, and thermoelastic deformation effect. Thermoelastic deformation caused by the temperature fluctuation of the electrode housing makes the local electrostatic force field at the test mass produce transient fluctuations, thus interfering with the measurement of acceleration. However, preliminary estimates indicate that the acceleration noise caused by this electrostatic fluctuation can be ignored [8]. The first three temperature effects are the main subjects of study. Schumaker et al. [9] outlined the potential sources of acceleration disturbances based on the LISA programs provided by the European Space Agency (ESA) and National Aeronautics and Space Administration (NASA), providing the rough minimum temperature disturbance requirements needed to meet the acceleration noise level. Carbone et al. [10] modeled an infinite flat plate based on the LISA Pathfinder, the pioneer satellite of the LISA program, and derived an equation relating temperature perturbation to acceleration noise with correction coefficients. Gibert Gutiérrez et al. [11] provided approximate contributions of each temperature perturbation on the acceleration noise at different pressures in the LISA Pathfinder. In summary, researchers mainly investigated the relationship between temperature perturbations and acceleration noise. However, obtaining accurate values for temperature perturbations is challenging due to the test masses being suspended in a vacuum environment, making direct measurement and precise calculation difficult. Additionally, the above noise analysis and estimation were based on studies from the LISA program and its pioneer satellite, LISA Pathfinder, which have obvious differences from the TianQin project in detection targets and inertial sensor design. Therefore, the following discussion focuses on the TianQin project, addressing the evaluation of the temperature perturbations and the acceleration noise caused by temperature disturbances.

Due to the limited number of temperature sensors onboard the spacecraft and the vacuum environment inside the electrode housing, it is challenging to directly obtain temperature disturbance values inside the electrode housing as input for noise estimation. To address this, a method is proposed that utilizes on-orbit experimental data and numerical simulation to obtain a complete temperature field. Based on this, a comprehensive acceleration noise evaluation scheme is also presented. This work not only provides a new approach for studying the impact of temperature disturbances on acceleration but also offers a basis for the thermal control design of the future TianQin mission.

The research process initially involves analyzing the relationship between temperature perturbation and acceleration noise existing in the inertial sensor of traditional heat pipe temperature control schemes, and providing the applicable noise relation equation. Secondly, an overall heat transfer model of the inertial sensor is established. In this model, the rarefied gas is equivalently simplified to a solid layer with low thermal conductivity. This allows for the calculation of the temperature difference signals between the opposite surfaces of the electrode housing. Then, the time frequency conversion is realized by using the low frequency band noise estimation spectral analysis algorithm (Logarithmic frequency axis Power Spectral Density, LPSD) to obtain the frequency domain temperature perturbation signals. This allows for the quantitative calculation of the impact of each temperature effect on the acceleration noise. Finally, it is compared with currently available on-orbit data, and thermal control design suggestions are given.

## 2. Theory and Methodology

### 2.1. Theoretical Modeling of Temperature Effect Noise

#### 2.1.1. Model Assumptions

**Figure 2:**
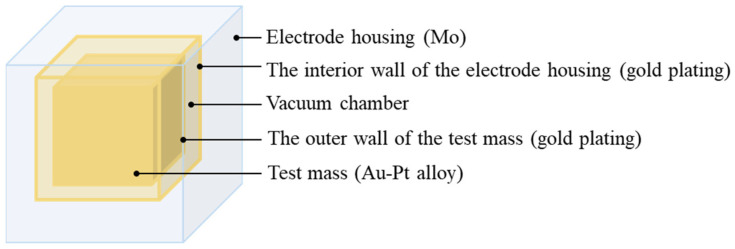
3D model of inertial sensor.

The actual inertial sensor includes complex structures such as front-end electronic equipment and high-precision positioning devices [12]. To facilitate analysis and calculation, as shown in Figure 2, the inertial sensor is simplified as a three-layer cubic structure, consisting of the electrode housing, the vacuum chamber, and the freely suspended test mass from the outside inwards. The electrode housing is a molybdenum metal cube with a side length of 80 mm and thickness of 10 mm, while the test mass is a gold–platinum alloy cube with a side length of 50 mm and a mass of 2.5 kg. The two are separated by a 5 mm vacuum cavity, designed to minimize the effect of gas flow on the acceleration measurement of the system. The interior wall of the electrode housing and the outer wall of the test mass are covered with a thin layer of gold plating of about 500 nm to reduce the surface emissivity to 0.02 and reduce the heat transfer from the vacuum layer to the test masses. When analyzing the influence of temperature effects, the two electrode housings on opposite sides are modeled as infinite flat plates, and the system average temperature of the inertial sensor is assumed to be $T_{0}$. With the high thermal conductivity of the electrode housing material and weak surface temperature perturbations (~$50\;mK/\sqrt{Hz}$), it can be assumed that temperature fluctuations on the surface are much smaller than $T_{0}$. In addition, since the thermal conductivity of the test mass material (314 W/(m·K)) is much higher than the equivalent thermal conductivity of the high vacuum insulating layer ($1.87\times 10^{-9}$ W/(m·K)), the test mass can be considered isothermal at $T_{0}$.

#### 2.1.2. Radiometer Effect

**Figure 3:**
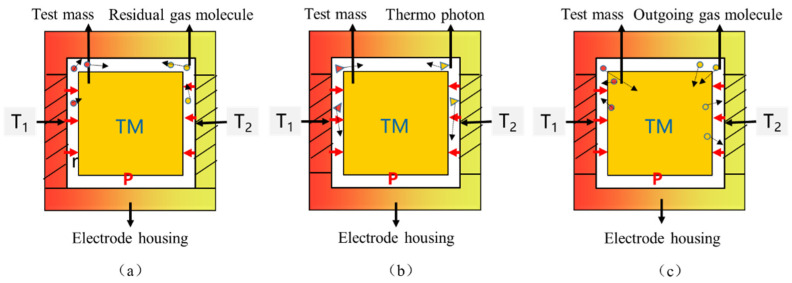
The principal diagrams of each effect: (**a**) Radiometer effect; (**b**) Radiation pressure effect; (**c**) Outgassing effect.

The radiometer effect is generated by gas molecular forces, which only appear in rarefied atmospheres. When there is a temperature gradient between the opposite sides of the electrode housing, the interaction between the gas molecules and the plate will cause the gas to flow from the hot side to the cold side. This flow generates a pressure difference that acts on the test mass and produces noise force. Figure 3a illustrates the action of the radiometer effect. The force resulting from the radiometer effect can be estimated using the theory of gas transpiration in the free molecular state. In the electrode housing, the test mass system, and the distance between the infinite plates can be considered negligible. When the two plates are at different average temperatures, $T_{1}$ and $T_{2}$, the approximate solution of the pressure between the two plates is:

(1) $$P_{pl}=\frac{P}{2}(\sqrt{\frac{T_{1}}{T_{0}}}+\sqrt{\frac{T_{2}}{T_{0}}})$$

where *P* is the pressure in the system, assuming a vacuum pressure of 10^−5^ Pa in the TianQin program; *T*_0_ is the average temperature of the system, set at 293 K. By integrating and linearizing this pressure on the two opposite surfaces of the test mass and introducing the correction coefficients, the acceleration resulting from the radiometer effect can be obtained, according to Newton’s second law, as [10]:

(2) $$a_{RM}=\frac{1}{4}\alpha_{RM}\frac{PA_{TM}}{m_{TM}T_{0}}\Delta T$$

where *α_RM_* is the correction factor for geometric and dimensional errors, with a value of 1.25 [10]. $A_{TM}$ is the area of the relative surface of the test mass, set at $2.5\times 10^{-3}\;m^{2}$; ΔT is the temperature difference between the relative surfaces; and *m* is the mass of the test mass, taking the value of 2.5 kg.

#### 2.1.3. Radiation Pressure Effect

The radiation pressure effect is generated by radiation pressure. When there is a temperature gradient between the opposite sides of the electrode housing, the momentum asymmetry of thermal photons emitted from the radiating surface leads to the asymmetry of the thermal radiation pressure on the surface of the test mass, thereby generating a noise force. Figure 3b illustrates the action of the radiation pressure effect. The radiation pressure force can be estimated by Boltzmann’s law. Assuming the infinite flat plate is a diffuse gray surface, the radiation pressure between two flat plates is [13]:

(3) $$P_{RP}=\frac{2\sigma }{3c}T_{0}^{4}$$

where σ is the Stefan–Boltzmann constant and c is the speed of light. By integrating and linearizing this pressure on the two opposite surfaces of the test mass, respectively, and introducing the correction coefficients, the acceleration generated by the radiation pressure effect can be obtained, according to Newton’s second law, as [10]:

(4) $$a_{RP}=\frac{8\sigma }{3c}\alpha_{RP}\frac{A_{TM}T_{0}^{3}}{m_{TM}}\Delta T$$

where $\alpha_{RP}$ is used to account for the additional effect observed in the torsional pendulum experiment at Trento University.

#### 2.1.4. Outgassing Effects

The outgassing effect is generated by surface outgassing pressure, where a surface exposed to a given pressure in the atmosphere undergoes particle exchange with the environment, causing molecules adsorbed on the inner surface of the electrode housing to escape. When a temperature gradient exists between the opposite surfaces of the electrode housing, the asymmetry of the outgassing rate of the test mass surfaces results in an asymmetry of pressure and generates a noise force. Figure 3c illustrates the action of the outgassing effect. Particle exchange depends strongly on temperature and particle species. At low pressures, the outgassing rate of molecules $Q_{rate}$ is defined by the outgassing prefactor $Q_{0}$ and the activation temperature $\Theta_{OG}$ [11]:

(5) $$Q_{rate}(T)=Q_{0}\exp(-\frac{\Theta_{OG}}{T})$$

Different particles have different outgassing rates, taking the average outgassing parameter as an approximation. Since the outgassing effect is suppressed by the resistance along the outgassing path from the test mass to the outgassing holes in the electrode housing, it is analogous to Ohm’s law U=IR. The pressure difference is equal to the flow rate divided by the flow conductance. Therefore, the pressure difference between the opposing surfaces is:

(6) $$\Delta P=\frac{\Delta Q_{rate}(T)}{C_{eff}}$$

where $C_{eff}$ is the equivalent flow conductance, including the flow conductivity of the path on both sides of the test mass and the hole in the electrode housing, taken as $4.3\times 10^{-2}\;m^{-3}/s$. After linearizing the force and temperature in the electrode housing, the relationship between the acceleration and the temperature difference on the opposite surfaces can be obtained as [10]:

(7) $$a_{OG}=A_{TM}\frac{Q_{rate}(T_{0})}{C_{eff}m_{TM}}\frac{\Theta_{OG}}{T_{0}^{2}}\Delta T$$

where $Q_{rate}$ is taken as 1.4 nJ/s and $\Theta_{OG}$ is taken as 30,000 K.

### 2.2. Numerical Simulation

#### 2.2.1. Heat Transfer Path Analysis

**Figure 4:**
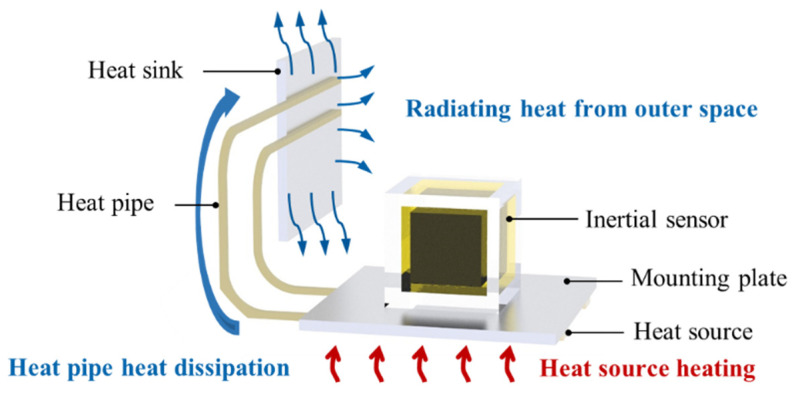
The overall model of the space inertial sensor.

The overall model of the inertial sensor and its thermal control system is shown in Figure 4. The inertial sensor is mounted on the mounting reference plate. During the acceleration measurement process, the relative position, velocity, and acceleration of the internal test mass of the inertial sensor will be interfered with by the temperature fluctuations and, thus, the stability will be reduced. Therefore, an active thermal control system is used to minimize the temperature variations. The working principle is as follows: when the temperature is high and needs to dissipate heat, the heat generated by the system is rapidly conducted to the heat sink through heat pipes affixed to the underside of the mounting plate, and then dissipated into outer space through radiative transfer. Conversely, when the temperature is low and needs to be heated, the heat source on the mounting plate is regulated by the relay to generate heat, which is transferred to the system through the mounting plate. Including the inertial sensor, the overall size of the whole thermal control and mechanical connection system is about 800 × 400 × 400 mm.

#### 2.2.2. Equivalent Heat Transfer Model for Rarefied Atmospheres

**Figure 5:**
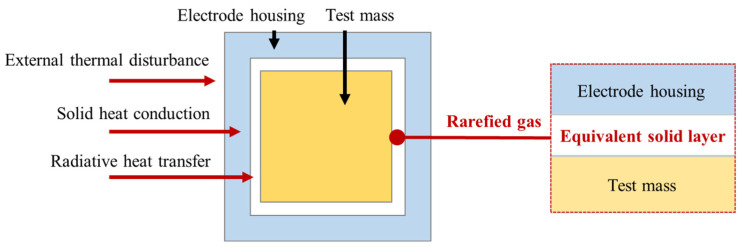
Equivalent heat transfer model of high vacuum layer.

In the vacuum chamber, heat transfer is dominated by the radiative heat transfer and thermal conductivity of the rarefied atmospheres in which they take place. However, the thermal conductivity of rarefied atmospheres is complex and involves small scale effects and a large number of parameters, which cannot be solved directly in simulations. Therefore, it is equated to a solid thermal conductivity layer with very low thermal conductivity, as shown in Figure 5. The required parameters are the thermal conductivity k, the density ρ, and the specific heat capacity at constant pressure $c_{p}$.

When the cavity vacuum is 10^−5^ Pa, the internal air is in a free molecular state, and its rarefied atmospheres thermal conductivity can be calculated from Equation (8) [14]:

(8) $$k=\frac{\gamma +1}{\gamma -1}\sqrt{\frac{R}{8\pi MT}}\cdot P\cdot \alpha \cdot \delta$$

In the formula, γ is the ratio of specific heat capacity at a constant pressure to specific heat capacity at a constant volume, which takes the value of 1.4 for air; *R* is the gas constant, which takes the value of 8.314 J/(mol·K); *M* is the molar mass, which is 29 g/mol for air; *T* is the average temperature inside the vacuum cavity, set at 293 K; *P* is the pressure inside the vacuum cavity; α is the thermal adaptation coefficient of gas, which is 0.85 for air [15]; δ is the gap between the two surfaces, which corresponds to the thickness of the vacuum cavity, set at 5 mm.

The ideal gas equation of state still applies in rarefied atmospheres, and thereby the density of the gas can be calculated from Equation (9):

(9) $$\rho =\frac{P}{RT}$$

Specific heat capacity at constant pressure refers to the amount of heat required to raise the temperature of a unit mass of a substance by 1 K under constant pressure. When the pressure drops to 10^−5^ Pa, the number of molecules per unit volume decreases, but the number of molecules per unit mass remains unchanged. As a result, the specific heat capacity at constant pressure of the near-vacuum layer is not significantly different from that observed at atmospheric pressure. In summary, the thermophysical parameters required for equating a rarefied gas to a solid are listed in Table 1.

#### 2.2.3. Thermal Simulation Methods

The finite element method is used to numerically simulate the heat transfer process of the overall thermal control system of the inertial sensor. To simplify the numerical calculations, the following assumptions are made: (1) a vacuum is maintained inside the inertial sensor, so that the convective heat transfer of the air can be neglected; (2) radiation heat transfer is neglected except with the vacuum cavity; and (3) all surfaces involved in radiation are assumed to be diffuse gray surfaces. Based on the above assumptions, the system can be simplified to a transient heat transfer process described by the differential equation for thermal conductivity (10) and the surface-to-surface radiation Equation (11):

(10) $$\rho c\frac{\partial T}{\partial t}=\nabla (k\nabla T)+q$$

where *ρ* is the density of the material, *c* is the specific heat capacity at a constant pressure of the material, *k* is the equivalent thermal conductivity, *T* is the temperature, and *q* is the body density per unit time of the heat source.

(11) $$q_{r}=J-G=\frac{\varepsilon }{1-\varepsilon }(E_{b}(t)-J)$$

where $q_{r}$ is the radiant heat flux density, J is the total radiant energy per unit time leaving the solid surface per unit area in units of $W/m^{2}$; G is the total radiant energy per unit time projected onto the solid surface per unit area in units of $W/m^{2}$; ε is the surface emissivity, which is set to 0.02 for the gold-plated surfaces in the model; and $E_{b}$ is the blackbody radiative forcing on the solid surface, which is a temperature-dependent function.

**Figure 6:**
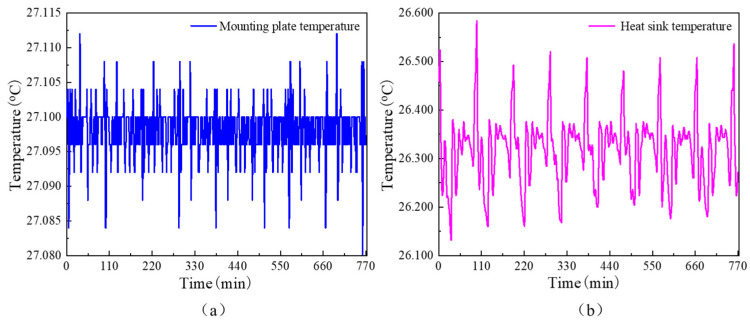
Temperature boundary data. (**a**) The measured data of the temperature of the installation plate. (**b**) The measured data of the temperature of the heat dissipation plate.

**Figure 7:**
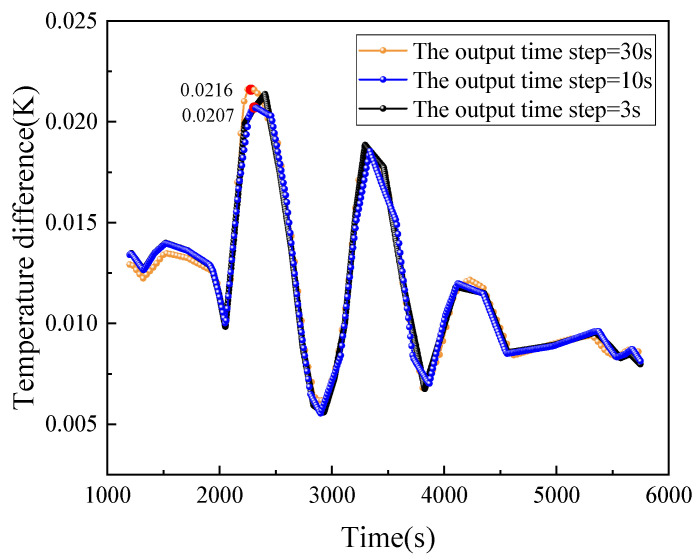
Time step verification.

The 3D model of the system is imported into the finite element simulation software and the material properties and thermophysical characteristics of each component are defined according to Table 2. On-orbit measured temperature data for the mounting plate and heat sink plate were given as boundary conditions, as shown in Figure 6. The temperature data is measured using a high-precision NTC (Negative Temperature Coefficient) thermistor with a measurement accuracy of 10^−3^ °C. The average temperature of the mounting plate fluctuates between 27.080 °C and 27.112 °C, while the average temperature of the heat dissipation plate fluctuates between 26.132 °C and 26.584 °C. The sampling time interval is approximately 60 s. A tetrahedral mesh is used for meshing and a grid independence test is performed, as shown in Table 3. To balance computational accuracy and resource efficiency, 1.22 million mesh elements are selected for the simulation. For the transient solver, time step tests for heat conduction are conducted using intervals of 30 s, 10 s, and 3 s, with the results shown in Figure 7. The maximum difference between the 30 s and 3 s intervals is approximately 0.0009 °C, which is reasonable when compared to the measurement accuracy of 0.001 °C for the actual data. Considering computational resources, the time step is set to 30 s, with a total simulation duration of 420 min. To improve computational accuracy, a smaller tolerance factor of 0.0001 is applied, and the residuals and solution results are displayed in real time.

### 2.3. Spectral Analysis Algorithm for Low Frequency Band Noise Estimation

**Figure 8:**
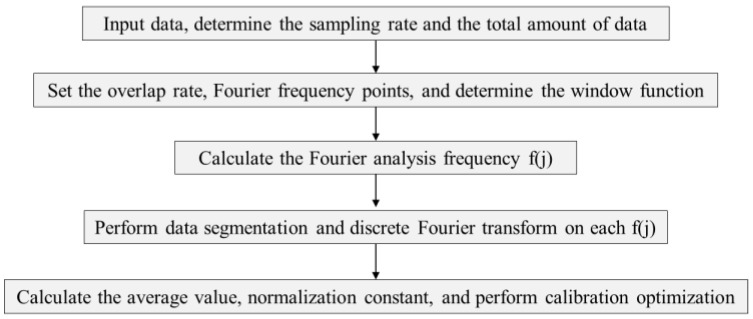
Flow chart of LPSD algorithm.

The measurement and evaluation of noise can usually be carried out in both the time and frequency domains. The time domain is mainly used to measure and collect gravitational wave and various noise signals. However, weak gravitational wave signals are often easily masked by noises, so it is necessary to extract, identify, and analyze these signals through the frequency domain [16]. In this paper, the Logarithmic frequency axis Power Spectral Density (LPSD) is used to analyze frequency domain characteristics. Compared to other methods, this noise assessment method adopts different frequency resolutions for different Fourier analysis frequencies and processes data in segments. This method can solve the problem of discontinuity in segmentation and burrs in high frequency bands in the case of large data volumes and wide bandwidths [17]. The specific steps of the LPSD algorithm are shown in Figure 8, which mainly include the selection of the logarithmic frequency, the segmentation of the data, and the normalization of the power spectrum.

According to the Nyquist sampling theorem [18], the frequency range requirement of the algorithm is:

(12) $$\frac{f_{s}}{N}\leq f_{\min}<f_{\max}\leq \frac{f_{s}}{2}$$

where $f_{s}$ is the sampling frequency, N is the total amount of data, $f_{\min}$ is the minimum value of the frequency range, and $f_{\max}$ is the maximum value of the frequency range. To meet the measurement bandwidth of 1 mHz~0.1 Hz of the TianQin program, the sampling rate of the temperature signal given by numerical simulation is 1/3 Hz and the total data is 8410.

## 3. Results and Analysis

### 3.1. Time Domain Temperature Analysis

**Figure 9:**
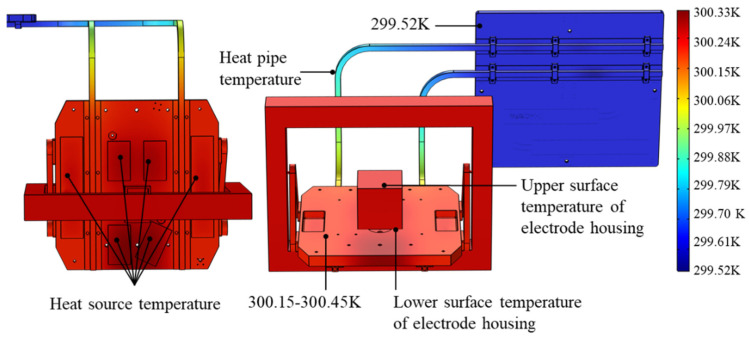
Temperature contour at the 420th minute.

Figure 9 shows the temperature contour of the spatial inertial sensor at the 420th minute. Due to the high thermal conductivity properties of the electrode housing material, the temperature difference between the upper and lower surfaces of the electrode housing is very small (~0.007 K). The temperature on the mounting plate is made very uniform by the thermal control system, and the overall temperature is about 300.15–300.45 K. The cooling plate, which represents the “low temperature zone” of the system, has a significantly lower temperature of approximately 299.52 K. The temperature on the heat pipe from the mounting plate side to the cooling plate side gradually decreases. Compared with the two plates, the temperature gradient on the heat pipe is more pronounced, resulting in more dramatic temperature changes. The heat on the mounting plate can be efficiently transferred to the cooling plate and dissipated via the high thermal conductivity heat pipe.

**Figure 10:**
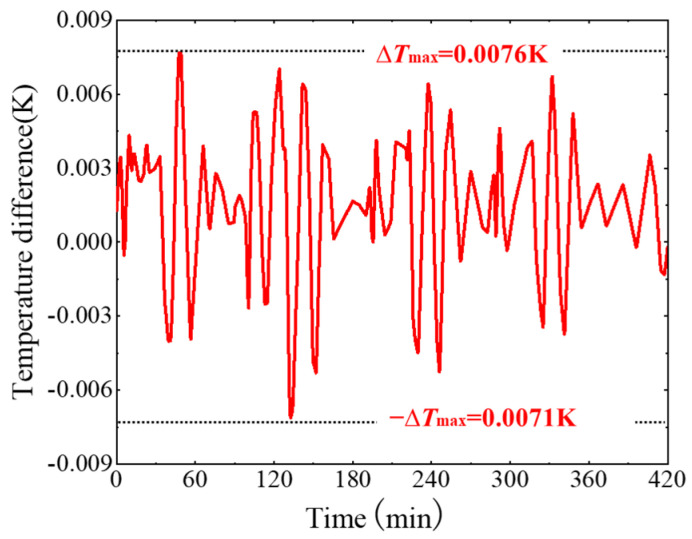
The signal diagram of the temperature difference between the relative surface of the electrode housing.

Figure 10 illustrates the temperature difference between the upper and lower relative surfaces of the electrode housing over a period of 0~420 min. The temperature difference curve obtained from the simulation calculation exhibits periodic fluctuations. Similar to the cyclic fluctuations of the temperature boundary, the period of the temperature difference fluctuation is 5750 s. From the extreme value range of the temperature data, the maximum temperature difference is 0.0076 K, which is much smaller than the fluctuation amplitude of the temperature boundary condition (~0.02 K). The attenuation of the amplitude is approximately 2.5 times, which indicates that after the active thermal control system, the temperature fluctuation amplitude is significantly reduced.

### 3.2. Frequency Domain Temperature Analysis

**Figure 11:**
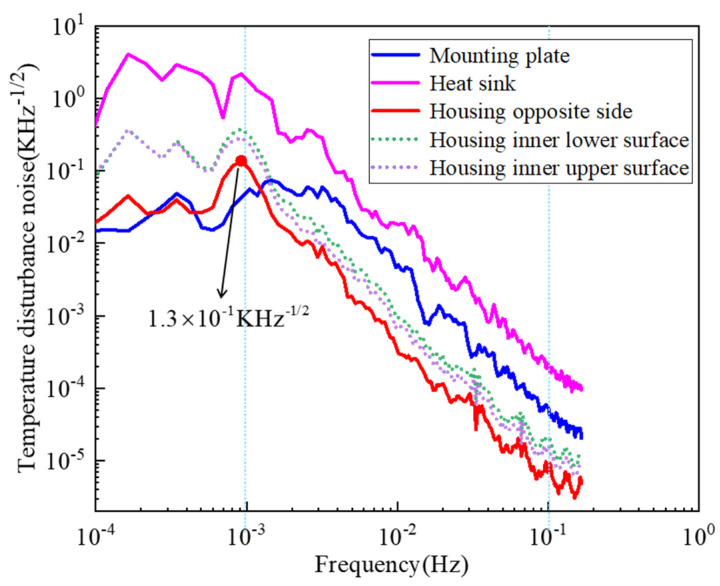
Power spectrum diagram of temperature disturbance.

The time domain temperature signals at each position of the structure are analyzed by the LPSD algorithm, and the power spectrum of each temperature perturbation can be obtained, as shown in Figure 11. It can be seen that the temperature perturbation power spectrum of the heat sink plate is the largest, indicating that its perturbation is the most violent. This is due to its participation in radiative heat transfer in outer space. In contrast, the mounting plate has better temperature stability compared to the heat sink plate after being regulated by the active thermal control system. The overall temperature perturbation of the electrode housing in the target bandwidth is generally smaller than that of the mounting plate. This difference arises because the measurement point of the mounting plate is on the lower surface while the electrode housing is located on the upper surface of the mounting plate. As heat conducts through the mounting plate, its own heat conduction and accumulation help to reduce the temperature perturbation. For the electrode housing, the temperature perturbation is higher on the lower surface than on the upper surface. This is because when the heat is transferred from the lower surface to the upper surface of the electrode housing, thermal conductivity and accumulation reduce the temperature perturbation.

The temperature difference signal of the relative surfaces of the electrode housing is essential for calculating acceleration noise. Its temperature difference power spectrum exhibits a downward trend within the measurement bandwidth. At the low frequency of 10^−3^ Hz, the temperature difference power spectrum reaches its peak, indicating that the energy of the temperature difference signal is strongest at this point, for $1.3\times 10^{-1}\;K/{\mathrm{Hz}}^{1/2}$. With the increase in frequency, the temperature difference signal energy gradually decreases, reaching approximately $10^{-5}\;K/{\mathrm{Hz}}^{1/2}$ at the high frequency boundary of 0.1 Hz. The upper limit of perturbation at low frequency is about four orders of magnitude higher than that at high frequency, indicating that the temperature perturbation at low frequency is more intense. Therefore, reducing the temperature fluctuation at low frequency is the key for the future thermal control design of the TianQin project.

### 3.3. Acceleration Noise Analysis

The relationship between each temperature effect and acceleration noise, along with the related parameters, are given in Equations (2), (4) and (7) in Section 2.1. The non-conservative force exerted on the test mass per K of temperature difference caused by each temperature effect at vacuum of $10^{-5}\mathrm{Pa}$ can be calculated and is noted as *β*, with units of pN/K, as shown in Table 4. It is evident that the outgassing effect causes the most interference, with *β* reaching 28.4 pN/K, followed by the radiometer effect (26.7 pN/K) and the radiation pressure effect (10.1 pN/K). Among the three effects, the radiometer effect is particularly sensitive to vacuum gas pressure. If the pressure inside the vacuum cavity is $10^{-6}$ Pa, which decreases by one order of magnitude compared to $10^{-5}\;\mathrm{Pa}$, its *β* will also decrease by one order of magnitude at this time, to only 2.67 pN/K, making it negligible compared to other temperature effects.

For the radiative pressure effect, since the vacuum cavity spacing is not negligible relative to the size of the test mass and this effect is related to the radiative properties of the surface, the coefficient $\alpha_{RP}$ is introduced to correct the equation. When surfaces are plated with gold to enhance their emissivity, thermal photons will impact more surfaces before being absorbed, resulting in a more uniform force on the test mass and effectively suppressing the radiative pressure effect. The outgassing effect is sensitive to the number and type of gas molecules, such as particles with low activation temperatures which have higher outgassing rates than those with high activation temperatures, making it so that the outgassing effect can be reduced by preheating the inertial sensors. In addition, if the venting device can exhaust as many gas molecules as possible that are present in the vacuum chamber, as well as those that are desorbed in the outgassing effect, the disturbances caused by temperature perturbations can be reduced.

**Figure 12:**
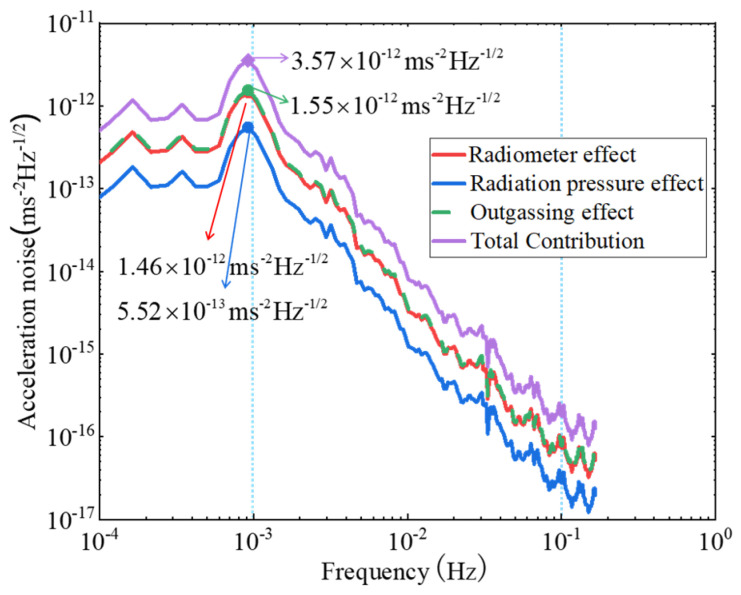
Power spectrum diagram of acceleration noise.

By substituting the temperature difference perturbation into the relational equation given by the previous analysis, the acceleration noise power spectrum can be calculated, as shown in Figure 12. The figure shows that outgassing has the largest effect on the acceleration noise, followed by the radiometer effect and the radiation pressure effect. The acceleration noise caused by all three effects shows a decreasing trend in the requested range. Similar to the decay of the temperature perturbation, this indicates that the acceleration noise is greater at low frequencies than at high frequencies. Notably, the total noise power spectrum amplitude is maximized at 10^−3^ Hz for $3.57\times 10^{-12}\;m/(s^{2}\cdot {\mathrm{Hz}}^{1/2})$.

### 3.4. Comparison with Existing On-Orbit Test Results

The results of this study are compared with the available on-orbit tests results, as shown in Table 5. The LISA Pathfinder satellite is the forerunner satellite of the LISA program, which was launched in 2015. Its on-orbit tests show that, under the temperature control of 10^−3^ K/Hz^1/2^ @1 mHz, the coefficient *β* representing the contribution of the temperature effect to the force is 20 pN/K, and the noise generated by the temperature perturbation to the acceleration is lower than $1\times 10^{-15}\;m/(s^{2}\cdot {\mathrm{Hz}}^{1/2})$ at 0.1 Hz [19]. TianQin-1, the first technology demonstration satellite of the TianQin project, was launched in 2019 [20]. The satellite’s residual acceleration, obtained from the first round of on-orbit tests, is about $1\times 10^{-10}\;m/(s^{2}\cdot {\mathrm{Hz}}^{1/2})$ at 0.1 Hz. Since in-depth research on the temperature effect of TianQin-1 has not been conducted, there is no clear test result on the influence of temperature disturbance on acceleration. In this study, the temperature control is 0.13 K/Hz^1/2^ @1 mHz, and the acceleration noise due to temperature perturbation is calculated to be $1.82\times 10^{-16}\;m/(s^{2}\cdot {\mathrm{Hz}}^{1/2})$ at 0.1 Hz, which is of a similar order of magnitude. The acceleration noise is the largest at 1 mHz for $3.57\times 10^{-12}\;m/(s^{2}\cdot {\mathrm{Hz}}^{1/2})$. There is a difference of more than three orders of magnitude compared to the requirement of $10^{-15}\;m/(s^{2}\cdot {\mathrm{Hz}}^{1/2})$ set by the TianQin program. Therefore, further improvement in temperature control is necessary. The theoretical analysis for the detection of gravitational waves in space requires that the ambient temperature control of the inertial sensors reaches 10^−5^ K/Hz^1/2^ orders of magnitude.

## 4. Conclusions

Based on the traditional heat pipe temperature control scheme, this paper analyzes the effect of temperature perturbation on the acceleration measurement of space inertial sensors. It establishes the relationship between temperature perturbation and acceleration noise, and calculates the acceleration noise caused by each temperature effect by combining numerical simulation and time-frequency conversion. The main conclusions are as follows:

(1) The acceleration noise generated by each temperature effect in the inertial sensor can be summarized as the product of the temperature perturbation power spectral density and the non-conservative force coefficient *β* (pN/K) of each effect, which is affected by both the temperature perturbation and the coefficient *β*.
(2) Based on the ideal state, where the coefficient *β* is taken as a constant, the relative contribution of each temperature effect on the acceleration noise is only related to *β*. The effects, ranked from largest to smallest, are the outgassing effect ($1.55\times 10^{-12}\;m/(s^{2}\cdot {\mathrm{Hz}}^{1/2})$), the radiometer effect ($1.46\times 10^{-12}\;m/(s^{2}\cdot {\mathrm{Hz}}^{1/2})$), and the radiation pressure effect ($5.52\times 10^{-13}\;m/(s^{2}\cdot {\mathrm{Hz}}^{1/2})$).
(3) The three kinds of acceleration noise show a decreasing trend in the requested range due to the temperature perturbation power spectral density, indicating that the temperature perturbations have a greater impact at low frequencies than at high frequencies. The total noise reaches its maximum at 10^−3^ Hz for $3.57\times 10^{-12}\;m/(s^{2}\cdot {\mathrm{Hz}}^{1/2})$, with a difference of more than three orders of magnitude compared to the TianQin target $10^{-15}\;m/(s^{2}\cdot {\mathrm{Hz}}^{1/2})$.
(4) Noise from the three effects can be attenuated by reducing the temperature perturbation or decreasing the value of *β*. For example, this can be achieved by reducing the pressure for the radiometer effect, enhancing the emissivity of the relevant surface for the radiation pressure effect, and discharging the gas molecules as much as possible through a drying device for the outgassing effect.

## Figures and Tables

**Table 1:** Equivalent thermophysical parameters for rarefied gas at $10^{-5}$ Pa.

Thermophysical Property	Value	Unit
*k*	1.8710 × 10^−9^	W/(m·K)
*ρ*	1.2 × 10^−10^	kg/m^3^
*c_p_*	1.004 × 10^3^	J/(kg·K)

**Table 2:** Thermophysical parameters of each component of the inertial sensor.

Component	Material	k W/(m·K)	ρ kg/m^3^	*c_p_* J/(kg·K)
Mounting plate and heat sink plate	7075 aluminum alloy	130	2810	960
Heat pipe	Copper ammonia heat pipe	20,000	8960	390
Heating pad	Silicon	16.5	2145	1136
Bracket	Titanium alloy	22	4510	530
Nail	Stainless steel	7850	44.5	475
Electrode housing	Molybdenum	138	10,200	250
Test mass	Gold-platinum alloy	314	20,000	130
Vacuum layer	Rarefied air	1.87 × 10^−9^	1.2 × 10^−10^	1004

**Table 3:** Grid independence verification.

Meshes	Temperature of Lower Surface of Electrode Housings (°C)	Temperature of Upper Surface of Electrode Housings (°C)	ΔT (°C)
620,744	27.1382	27.1351	0.0031
1,225,535	27.1439	27.1426	0.0013
1,458,842	27.1452	27.1439	0.0013
1,568,981	27.1467	27.1454	0.0013

**Table 4:** *β* at $10^{-5}$ Pa.

Temperature Effect	*β* (pN/K)
Radiometer effect	26.7
Radiation pressure effect	10.1
Outgassing effect	28.4
Total temperature effect	65.2

**Table 5:** The comparison between the existing on-orbit test results and present results.

Source	Temperature Control LEVEL @1 mHz	Acceleration Noise Value @0.1 Hz
LISA Pathfinder	$10^{-3\;}K/{\mathrm{Hz}}^{1/2}$	$1\times 10^{-15}\;m/(s^{2}\cdot {\mathrm{Hz}}^{1/2})$
TianQin-1	$0.025\;K/{\mathrm{Hz}}^{1/2}$	$1\times 10^{-10}\;m/(s^{2}\cdot {\mathrm{Hz}}^{1/2})$
Present results	$0.13\;K/{\mathrm{Hz}}^{1/2}$	$1.82\times 10^{-16}\;m/(s^{2}\cdot {\mathrm{Hz}}^{1/2})$

## Data Availability

Some or all data, models, or code that support the findings of this study are available from the corresponding author upon reasonable request.
